# Supplementary material for: Clinical Relevance of Tumour-Infiltrating Immune Cells in HER2-Negative Breast Cancer Treated with Neoadjuvant Therapy
Source: Int J Mol Sci. 2024 Feb 23;25(5):2627. doi: 10.3390/ijms25052627 (PMC10932208; doi:10.3390/ijms25052627)
Supplement: Supplementary file 1 [file ijms-2832853-supplementary.pdf › ijms-2832853-supplementary.pdf]

**Table S1.** Univariate analyses of sTILs and their association with clinicopathological features.

| | | **sTILs Continuous Variable** | | **sTILs Stratified Variable** | |
|---|---|---|---|---|---|
| | | OR (95% CI ) | *p* | OR (95% CI ) | *p* |
| Tumor size | (T2/T3/T4 vs T1) | 1.09 [0.91;1.31] | 0.354 | 2.32 [0.64;8.40] | 0.212 |
| Histologic grade | (G3 vs G1/G2) | 0.86 [0.76;0.98] | **0.023** | 0.45 [0.20;1.02] | 0.058 |
| Lymphovascular invasion | (Positive vs Negative) | 1.01 [0.86;1.20] | 0.882 | 1.27 [0.48;3.39] | 0.642 |
| Perineural invasion | (Positive vs Negative) | 0.99 [0.80;1.23] | 0.945 | 0.80 [0.22;2.92] | 0.745 |
| ki-67 index | (≥14% vs <14%) | 0.88 [0.76;1.02] | 0.088 | 0.49 [0.23;1.06] | 0.072 |
| Clinical N-stage | (N+ vs N0) | 0.97 [0.86;1.10] | 0.688 | 0.94 [0.45;1.95] | 0.867 |
| Hormonal receptor status | (Negative vs Positive) | 0.90 [0.79;1.03] | 0.129 | 0.47 [0.20;1.13] | 0.096 |

sTILs: stromal tumor-infiltrating lymphocytes, sTILs stratified variable: high-TILs (> 10%) vs low-TILs (≤ 10%), OR: odds ratio, CIs: confidence intervals. The statistical parameters shown in the table refer to sTILs (continuous and stratified variables). Statistically significant *p*-values are marked in bold.

**Table S2.** sTILs and their association with systemic biomarkers.

| | **sTILs Continuous Variable** | | **sTILs Stratified Variable** | | |
|---|---|---|---|---|---|
| | | | **High-sTILs** | **Low-sTILs** | |
| | Spearman's rho | *p* | median (Q1, Q3) | median (Q1, Q3) | *p* |
| MDSC (Entire cohort) | -0.277 | **0.039** | 1.4 (1.1-2.2) | 2.0 (1.5-2.5) | **0.031** |
| MDSC (Luminal) | -0.311 | **0.038** | 1.4 (1.1-1.7) | 2.1 (1.5-2.6) | **0.037** |
| exosomes (Entire cohort) | -0.293 | **0.035** | 8.7 (6.6, 18.0) | 14.1 (7.5, 23.7) | 0.154 |
| exosomes (Luminal) | -0.255 | 0.103 | 9.3 (6.2, 18.3) | 11.7 (6.8, 21.7) | 0.525 |

sTILs: stromal tumor-infiltrating lymphocytes, MDSC: myeloid-derived suppressor cells. TILs stratified variable: high-TILs (> 10%) vs low-TILs (≤ 10%). Statistically significant *p*-values are marked in bold.
